# Sucrose as a key nutritional marker distinguishing vegetable and grain soybeans, regulated by *GmZF-HD1* via *GmSPS17* in seeds

**DOI:** 10.1093/hr/uhaf242

**Published:** 2025-09-15

**Authors:** Changkai Liu, Qiuying Zhang, Yanfeng Hu, Yansheng Li, Xiaobing Liu

**Affiliations:** State Key Laboratory of Black Soils Conservation and Utilization, Northeast Institute of Geography and Agroecology, CAS, Harbin 150081, China; State Key Laboratory of Black Soils Conservation and Utilization, Northeast Institute of Geography and Agroecology, CAS, Harbin 150081, China; State Key Laboratory of Black Soils Conservation and Utilization, Northeast Institute of Geography and Agroecology, CAS, Harbin 150081, China; State Key Laboratory of Black Soils Conservation and Utilization, Northeast Institute of Geography and Agroecology, CAS, Harbin 150081, China; State Key Laboratory of Black Soils Conservation and Utilization, Northeast Institute of Geography and Agroecology, CAS, Harbin 150081, China

## Abstract

Vegetable and grain soybeans are typically distinguished by harvest time and pod size, yet their nutritional differences are often overlooked in breeding programs. This study compared 10 varieties each of vegetable and grain soybeans to find key nutritional markers distinguishing them. Results showed that vegetable soybeans have higher concentrations of sucrose, total soluble sugar, and crude protein, along with lower concentrations of crude oil and total fatty acid. Specifically, vegetable soybeans contain a relatively higher amount of unsaturated fatty acids, particularly oleic acid, at green edible stages. Principal component analysis of 12 nutritional components revealed clear distinctions between vegetable and grain soybeans. Additionally, machine learning algorithms identified sucrose as the most critical nutritional marker for distinguishing these two types. Dynamic RNA-seq analysis combined with weighted gene co-expression network analysis identified a sucrose-related module, highlighting *GmSPS17* as a predominant sucrose phosphate synthase encoding gene involved in sucrose accumulation in soybean seeds. Furthermore, we identified *GmZF-HD1* as an upstream transcription factor regulating *GmSPS17.* Yeast one-hybrid, luciferase, and electrophoretic mobility shift assays confirmed that *GmZF-HD1* directly activates *GmSPS17* transcription. Overexpression experiments in hairy roots validated that *GmZF-HD1* enhances *GmSPS17* expression, thereby increasing sucrose accumulation. In summary, this study establishes sucrose as a key nutritional marker for distinguishing vegetable soybeans from grain soybeans and elucidates the *GmZF-HD1*–*GmSPS17* regulatory pathway, providing valuable insights into sugar accumulation mechanisms and offering guidance for breeding high-sugar vegetable soybean varieties.

## Introduction

Soybeans, a globally significant crop, can be categorized into different specialty types based on their intended uses. The main types of soybeans are grain soybeans and vegetable soybeans. Grain soybeans are primarily produced for soy-food processing, oil extraction, and animal feed. In contrast, vegetable soybeans, known as ‘maodou’ in China and ‘edamame’ in Japan, are harvested during the fresh pod stage, when the seeds have filled to over 80% of the pod’s length [1]. With the growing demand for nutritious food among health-conscious consumers, vegetable soybeans are gaining popularity in global diets.

Despite the growing recognition of specialized vegetable soybean varieties, a significant number of common large-seeded soybeans are still harvested fresh and used as vegetable soybeans. This practice hinders the development and promotion of specialized vegetable soybean varieties and may cause confusion among consumers. Moreover, some individuals argue that vegetable soybeans can be used as grain soybeans under certain conditions, due to their high nutritional quality, including high levels of protein, free amino acids, and unsaturated fatty acids (UFAs) [2–5]. Given these overlapping uses and potential for confusion, it is necessary to clearly define and distinguish vegetable soybeans from grain soybeans based on nutritional components [6].

Saldivar *et al.* [7] proposed that vegetable soybeans should exhibit a high protein content (exceeding 45%) and a low oil content (below 18%). This recommendation is supported by Sonkamble *et al.* [4], who found that vegetable soybeans are nutritionally superior to grain types in terms of protein, carbohydrate, sugar, iron, and zinc content. Furthermore, Zhang *et al.* [8] identified sucrose as a critical determinant of vegetable soybean’s eating quality. To optimize breeding efforts, it is essential to adopt a targeted approach that prioritizes these key nutritional traits. This strategy would enable breeders to develop soybean varieties that not only meet the criteria for classification as vegetable soybeans but also provide enhanced nutritional benefits, thus appealing to consumers [4]. Such a focused breeding approach will streamline the development process and ensure that the end product is clearly differentiated from grain soybeans, catering to the specific needs of the market and consumers.

Machine learning (ML), a crucial branch of artificial intelligence (AI) technology, has been increasingly applied in crop breeding [9, 10]. The application of ML in breeding provides powerful tools that not only enhance breeding efficiency but also help breeders develop varieties that better meet market demands [11]. Beyond yield prediction, ML is instrumental in identifying and selecting varieties with high nutritional value [12]. For instance, by analyzing the protein, oil, and carbohydrate content of soybeans, ML can assist breeders in selecting varieties with high protein and low oil content [13]. Additionally, ML is used to integrate omics data, thereby improving the understanding of plant genetic diversity and facilitating the connection between genotype and phenotype [14, 15]. Therefore, utilizing ML to predict and differentiate key nutritional components that distinguish vegetable soybeans from grain soybeans is both a feasible and effective approach.

Protein and soluble sugar (mainly sucrose) contents are the primary nutritional components used to evaluate the nutritional quality of vegetable soybeans [16]. Several regulatory genes involved in protein accumulation, such as B3 family transcription factors (TFs) like LEC2, ABI3, FUS3, and HAP3 family CCAAT-box-binding factor LEC1, have been identified. These genes form the LAFL TF network, which is essential for seed development and protein accumulation [17]. Similarly, TFs from the Dof, bZIP, and MYB families also play significant roles in lipid and protein accumulation [18]. In parallel, there is a growing focus on identifying genes related to soybean sugar content, as sugars influence both the taste of vegetable soybeans and the flavor of soybean products [19]. Key genes in the sugar metabolism pathway, such as sucrose phosphate synthase (SPS) and sucrose synthase (SUS), as well as those involved in sucrose metabolism and transport, including hexokinase (HK), fructokinase (scrK), invertase (INV), Sugars Will Eventually be Exported Transporters (SWEET), and sucrose transporter (SUT/SUC), have been identified [19, 20]. Additionally, TFs from AP2/ERF, HSF, and ARF families are associated with sucrose accumulation [21]. These findings enhance our understanding of the nutritional quality and metabolic processes in soybeans, particularly the interplay between sugar content and protein synthesis, and their combined impact on product quality [22, 23].

Leveraging the nutritional benefits of vegetable soybeans, this study compared their nutritional profiles with those of grain soybeans to identify key differences. Using machine learning, we pinpointed sucrose as a critical marker distinguishing vegetable from grain soybeans. Further, dynamic transcriptome analysis and weighted gene co-expression network analysis (WGCNA) [24] revealed regulatory modules and candidate genes linked to sucrose accumulation, with *GmSPS17* and its regulator *GmZF-HD1* being characterized through molecular assays. These insights offer valuable guidance for breeding high-quality vegetable soybean varieties.

## Results

### Nutritional components comparison between vegetable soybeans and grain soybeans

**Figure 1:**
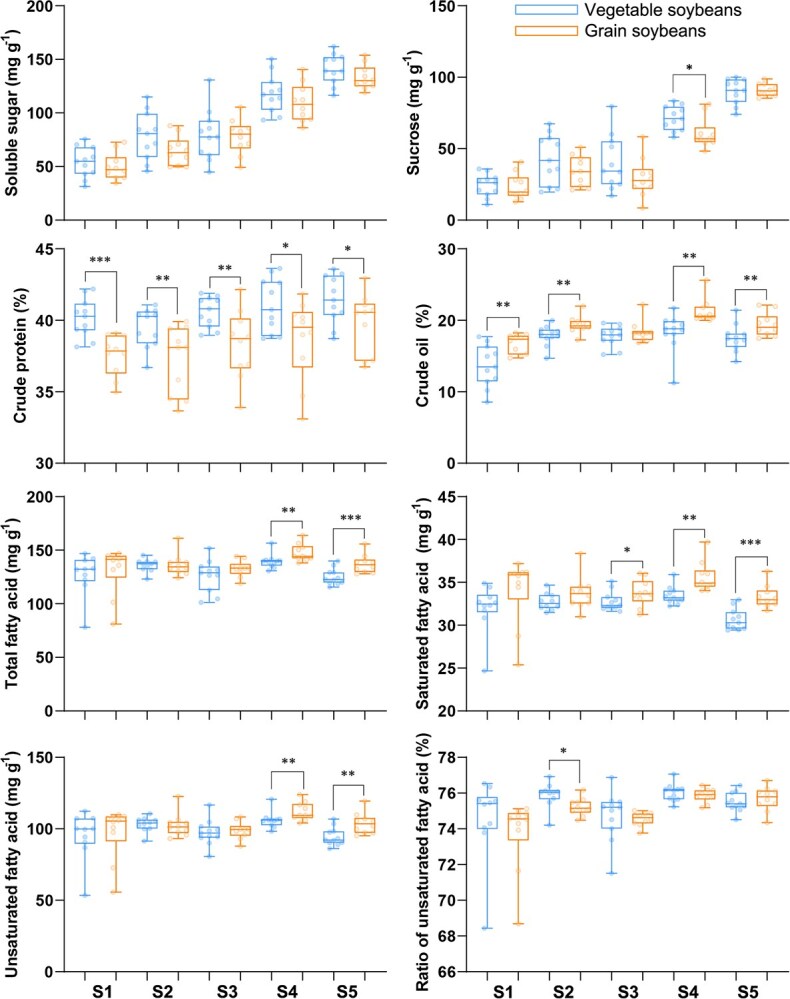
Comparison of nutritional compositions between vegetable soybean and grain soybean during seed development. Growth stages: S1 (5 days after R5, beginning seed stage), S2 (10 days after R5), S3 (15 days after R5; R6, full seed stage), S4 (20 days after R5), and S5 (R8, full maturity stage). The box plot shows the mean values of three biological replicates for each of the 10 vegetable soybean (VS) varieties and 10 grain soybean (GS) varieties. An independent samples *t*-test was conducted to assess the significance of differences between the two groups. The significance levels are indicated as follows: ^*^P < 0.05, ^**^P < 0.01, and ^***^P < 0.001 .

The box plot was constructed to illustrate variations in the nutritional compositions between vegetable soybeans and grain soybeans across five seed development stages (Fig. 1). Generally, compared to grain soybeans, vegetable soybean varieties exhibited higher concentrations of total soluble sugar, sucrose, and crude protein. The higher ratio of UFAs to total fatty acids (TFAs) was found in vegetable soybeans, particularly prior to the S4 stage. Conversely, lower concentrations of crude oil, TFAs, and saturated fatty acids (SFAs) were observed. More details are provided below.

### Soluble sugar and sucrose

The concentration of soluble sugar and sucrose in vegetable soybean varieties was, on average, 5.8% and 9.0% higher, respectively, than in grain soybeans across the five developmental stages. From S1 (R5.5 stage) to S5 (R8 stage), the rate of increase in soluble sugar and sucrose concentrations was similar in both soybean types. The soluble sugar and sucrose concentrations increased by 1.75- and 2.86-fold in vegetable soybeans and by 1.67- and 2.88-fold in grain soybeans. Additionally, as the seeds developed, the coefficients of variation (CVs) for soluble sugar and sucrose in both types of varieties underwent a substantial decline, decreasing from 25% to 9.5% for soluble sugar and from 35.4% to 7.3% for sucrose (Supporting Information Dataset S1).

### Crude protein and oil

From stage S1 to S5, the mean crude protein concentration in vegetable soybean varieties was 6.4% higher than in grain soybean varieties. The mean crude protein concentration increased by 3.3% in vegetable soybean varieties and 5.9% in grain soybean varieties. Throughout the seed development stages, vegetable soybeans exhibited lower CVs compared to grain soybean varieties, especially at the S3 stage, where the CV was only 2.4% in vegetable soybean varieties but 6.3% in grain soybean varieties (Supporting Information Dataset S1).

The S4 stage was characterized by the highest concentration of crude oil, with a grain soybean variety reaching a peak concentration of 26%, while a vegetable soybean variety had the lowest concentration of 11%. From S1 to S4, the mean crude oil concentration increased by 35% in vegetable soybeans and 27% in grain soybeans. Additionally, the mean crude oil concentration during seed development in vegetable soybean varieties was 12% lower than that in grain soybean varieties.

### Fatty acids

**Figure 2:**
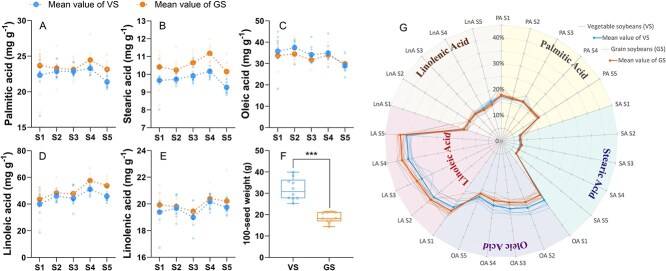
Comparison of fatty acid components (A–E), proportion of each component relative to TFA content (G), and 100-seed weight (F) in vegetable soybeans and grain soybeans during seed development. The box plot for 100-seed weight (F) shows the mean values of three biological replicates for each of the 10 vegetable soybean (VS) varieties and 10 grain soybean (GS) varieties. An independent samples *t*-test was conducted for 100-seed weight (F) to assess the significance of differences between the two groups, with the significance level indicated as ^***^P < 0.001.

Similar to crude oil, the highest concentration of TFAs occurred at the S4 stage with a mean value of 137.9 mg/g in vegetable soybeans and 147.7 mg/g in grain soybeans (Fig. 1). Over the five developmental stages, the mean TFA concentration in vegetable soybean varieties was 5.2% lower than that in grain soybean varieties. However, the mean concentration of SFAs (palmitic acid and stearic acid) was 5.8% higher in grain soybean varieties than in vegetable soybean varieties across the same stages. Pronounced differences in UFA concentrations (oleic acid, linoleic acid, and linolenic acid) between vegetable soybean and grain soybean varieties were observed after the S3 stage. However, the ratio of UFAs to TFAs was higher in vegetable soybeans before the S4 stage, primarily due to the relatively higher concentration of oleic acid in vegetable soybean varieties (Fig. 2C).

As seed development progressed, the concentrations of palmitic acid, stearic acid, linoleic acid, and linolenic acid peaked at the S4 stage, and their concentrations were generally higher in grain soybean varieties than in vegetable soybean varieties (Fig. 2). In contrast, the highest concentration of oleic acid was at the S2 stage, while the lowest oleic acid concentration was at the S5 stage. Among the five fatty acid components, linoleic acid accounted for the majority of the TFAs, and stearic acid comprised the least proportion (Fig. 2G). Generally, the relative proportion of linoleic acid and stearic acid was higher in grain soybeans, whereas the relative content of linolenic acid and oleic acid was higher in vegetable soybeans.

### 100-seed weight and correlation among nutritional qualities

Significant differences in 100-seed weight were observed between vegetable soybean varieties and grain soybean varieties (Fig. 2F). On average, the 100-seed weight of vegetable soybeans was 31.9 g, which was 72% higher than that of grain soybeans (18.6 g). The highest recorded 100-seed weight was 39.8 g in vegetable soybeans, while the lowest was 14.4 g in grain soybeans.

**Figure 3:**
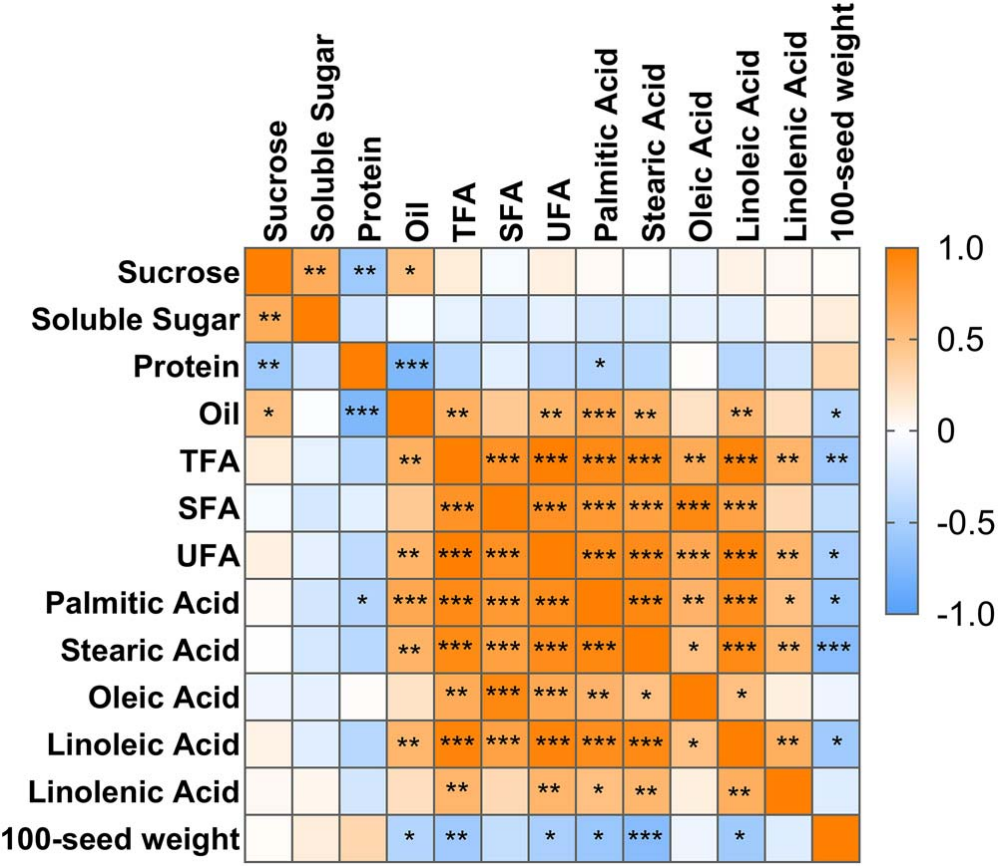
Correlation matrix of nutritional traits analyzed using the whole data set. The star markers (*, **, ***) indicate the significance levels of the correlations, which were determined using Pearson correlation tests. The significance levels are defined as ^*^P < 0.05, ^**^P < 0.01, and ^***^P < 0.001.

Sucrose concentration was positively correlated with soluble sugar concentration but negatively correlated with crude protein concentration (Fig. 3). Additionally, crude protein concentration was negatively correlated with crude oil and palmitic acid concentrations. The concentration of crude oil was also positively correlated with TFAs, SFAs, and UFAs. The 100-seed weight was negatively correlated with the concentration of crude oil, TFA, SFA, UFA, palmitic acid, stearic acid, and linoleic acid.

### Identification of key nutritional biomarkers for distinguishing vegetable soybeans from grain soybeans

**Figure 4:**
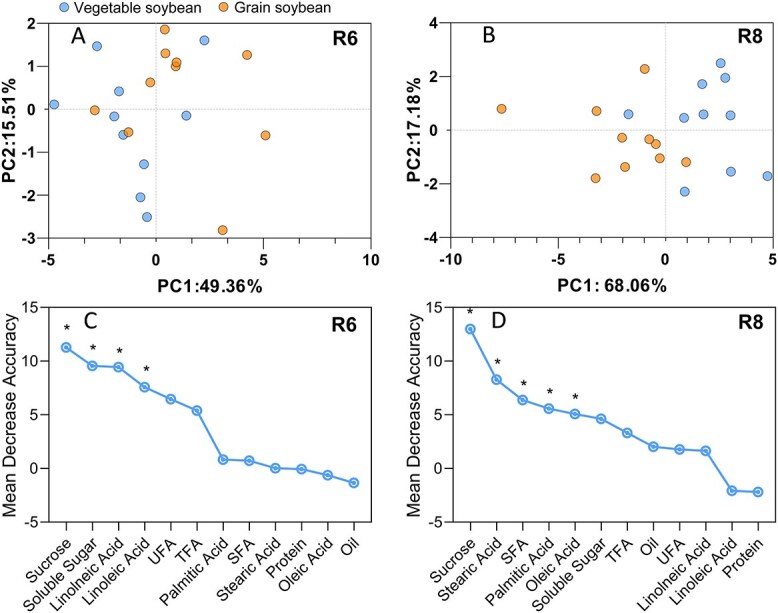
PCA of vegetable soybeans and grain soybeans at R6 (A) and R8 (B) stages, and classification index analysis based on machine learning (Random Forest algorithm) at R6 (C) and R8 (D) stages. Significance levels of the variable importance scores, determined using the mean squared error from permutation tests, are defined as ^*^P < 0.05.

Since the pod-filling stage (R6) and fully mature stage (R8) are the respective critical harvesting periods for vegetable soybeans and grain soybeans, principal component analysis (PCA) and random forest analysis were conducted separately using the nutritional components at R6 and R8 to identify key indicative quality traits that differentiate vegetable soybeans from grain soybeans (Fig. 4).

PCA effectively separated the two types of soybeans. The analysis of nutritional indicators revealed a clear separation along the first component (PC1), allowing for differentiation between vegetable soybeans and grain soybeans at both R6 and R8 stages. At R6 stage (Fig. 4A), PC1 and PC2 accounted for 49% and 16% of the total variation, respectively. Similarly, at R8 stage (Fig. 4B), PC1 and PC2 explained 68% and 17% of the total variation, respectively.

A comprehensive set of 12 nutritional components, namely sucrose, soluble sugar, crude oil, crude protein, TFAs, UFAs, SFAs, and five fatty acid components, was utilized for constructing the random forest model. The relative importance of these selected nutritional components, as determined by the mean decrease accuracy (MDA) value in the random forest model, is shown in Fig. 4C for the R6 stage and Fig. 4D for the R8 stage. At R6 stage, sucrose, soluble sugar, linoleic acid, and linolenic acid were identified as significant factors for distinguishing vegetable soybeans from grain soybeans. Similarly, at R8 stage, sucrose, stearic acid, SFAs, palmitic acid, and oleic acid were identified as significant factors for distinguishing vegetable soybeans from grain soybeans. Overall, sucrose was consistently identified as the key indicator for differentiating vegetable soybeans from grain soybeans.

To further investigate the role of sucrose as a key indicator, we analyzed the sucrose concentrations of 30 vegetable soybean varieties and 30 grain soybean varieties. This analysis confirmed the results from the random forest model, demonstrating that vegetable soybeans have a significantly higher sucrose content than grain soybeans (Fig. S1).

### Genetic basis of dynamic changes in nutritional assessment indicators between vegetable and grain soybeans

To further examine the genetic basis of the nutritional assessment indicators that distinguish vegetable soybeans from grain soybeans, seven soybean varieties were selected at three stages for RNA-Seq analysis. These varieties included three vegetable soybeans and four grain soybeans. After removing adapter reads, ambiguous reads, and low-quality reads, an average of 6.05 Gb of clean bases was obtained (Supporting Information Dataset S2). To estimate the accuracy and representativeness of the data, the expression of housekeeping genes was analyzed [25, 26]. The consistent expression of 452 housekeeping genes across all three developmental stages (Fig. S2) confirmed the reliability of the transcriptomic data.

**Figure 5:**
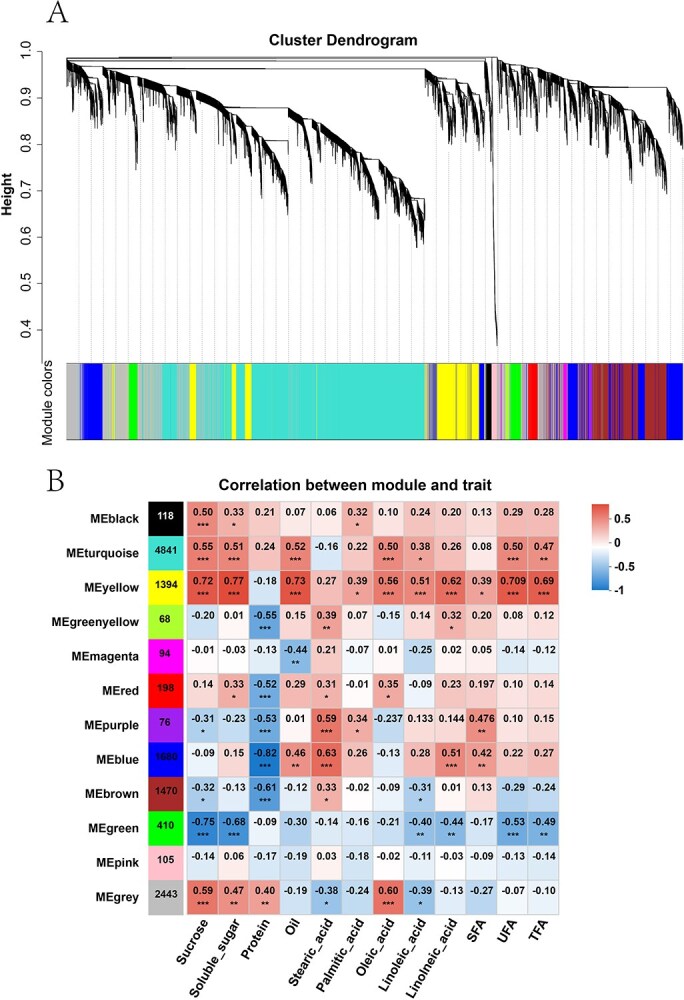
Co-expression network (A) and correlations (B) between module eigengene (ME) and nutritional components in soybean varieties, using Pearson correlation analysis. The significance levels are defined as ^*^P < 0.05, ^**^P < 0.01, and ^***^P < 0.001.

To gain further insight into the regulation of nutrient changes throughout soybean seed development, WGCNA was performed. Through this process, the initial set of 57 664 genes was filtered, retaining 12 897 genes that exhibited sufficient expression levels and variability for further analysis. A total of 12 co-expression modules were identified based on their similar expression patterns (Fig. 5A; Supporting Information Dataset S3).

The heatmap displaying module–trait correlations (Fig. 5B) revealed that genes within the turquoise and yellow modules were positively correlated with sucrose, soluble sugars, oil, and the majority of fatty acids. In contrast, the green module was negatively correlated with sucrose and soluble sugars. Additionally, the blue module was positively correlated with protein content.

### Generation of sucrose metabolic regulatory networks

**Figure 6:**
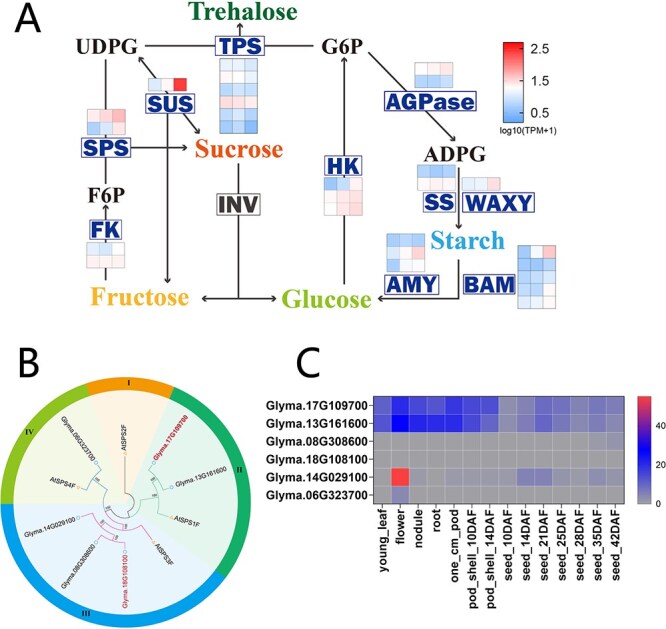
Analysis of gene expression and phylogenetics in soybean. (A) Expression profiles of genes in turquoise and yellow WGCNA modules associated with metabolic pathways for soluble sugars. (B) Phylogenetic analysis of SPS proteins. (C) Expression patterns of the soybean *SPS* family genes across various tissues and seed development stages based on SoyBase website data.

The correlation analysis revealed a strong positive correlation (0.755) between the turquoise and yellow modules (Supporting Information Dataset S4). Because these modules clustered together, the genes from the turquoise and yellow modules were combined and further screened for candidate genes associated with sucrose metabolism. Through this process, 27 genes were identified as involved in sucrose metabolism (Fig. 6A), including two *SPSs*, one *SUS*, two *fructokinases* (*FK*), three *hexokinases* (*HK*), eight *alpha-*/*beta-amylases* (*AMY*/*BAM*), one *waxy gene* (*WAXY*), two *starch synthases* (*SS*), two *ADP-glucose pyrophosphorylases* (*AGPase*), and *six trehalose-6-phosphate synthases* (*TPS*). The expression of these genes was highly correlated with the accumulation of sucrose (Supporting Information Dataset S5).

Among the 27 candidate genes, SPS and SUS are considered pivotal for sucrose biosynthesis. SPS catalyzes the rate-limiting step of sucrose synthesis, whereas SUS functions bidirectionally in both sucrose synthesis and degradation [27]. To further explore their roles in sucrose accumulation, we used quantitative real-time PCR (qRT-PCR) to examine the dynamic expression patterns of two highly expressed genes within the module, *Glyma.17G109700* (*GmSPS17*) and *Glyma.15G151000* (*GmSUS15*), across four developmental stages of seeds in 10 vegetable soybean (VS) varieties and 10 grain soybean (GS) varieties (Fig. S3). The results showed that the expression pattern of *GmSPS17* was more consistent with the dynamic trend of sucrose accumulation in seeds, with a higher correlation coefficient (0.55, *P* < 0.001), while *GmSUS15* exhibited a lower correlation (0.42, *P* < 0.001) (Fig. S3). Given that SUS is a bidirectional regulatory enzyme for sucrose metabolism and may perform different functions at various stages of seed development, we focused further investigation on *GmSPS17* in this research.

Phylogenetic analysis demonstrated that the soybean SPS family genes, *GmSPS17* and *Glyma.13G161600*, share a close evolutionary relationship with the *Arabidopsis AtSPS1F* gene, as evidenced by their clustering together (Fig. 6B). Utilizing transcriptome data from the SoyBase website (https://legacy.soybase.org/), a detailed tissue-specific expression analysis of the soybean SPS family genes was performed. The analysis revealed that *GmSPS17* and *Glyma.13G161600* display analogous expression profiles, with both genes being actively transcribed across diverse tissues of the soybean plant (Fig. 6C). Notably, these genes were particularly highly expressed in soybean seeds, indicating their significance within the seed’s *SPS* gene repertoire. Since *GmSPS17* is significantly and positively correlated with sucrose in the combined module, we then focused on its expression regulation pattern.

### Transcriptional regulation of *GmSPS17*

Given the differential expression of *GmSPS17* observed across various soybean varieties, we sequenced and compared the 2000-bp promoter regions of this gene in 32 soybean varieties. Six single nucleotide polymorphisms (SNPs) were identified within the promoter region (Fig. S4), predominantly located within 400-bp upstream of the transcription start site (Supporting Information Dataset S6). Using the PlantCARE online tool for prediction, these SNPs were found to be associated with several key *cis*-acting elements, including the TATA-box, CAAT-box, and WUN-motif (Supporting Information Dataset S7). Notably, a T-to-G mutation at the physical position 8 599 257, which is enriched in vegetable soybeans, was detected within the TATA-box element (Supporting Information Datasets S6 and S7). This mutation may positively influence the transcriptional expression of the *GmSPS17* gene in vegetable soybeans, although this hypothesis requires further experimental validation.

To gain a deeper understanding of the transcriptional regulatory mechanisms governing *GmSPS17*, the yeast one-hybrid screening system was employed. This screening yielded a total of 105 positive clones, which were then individually sequenced. Following BLAST alignment analysis and the elimination of redundant sequences, 56 unique protein-encoding genes were identified (Supporting Information Dataset S8). Among these, *Glyma.01G025000* (*GmZF-HD1*), which encodes a Zinc Finger Homeodomain (ZF-HD) TF and is connected to the WGCNA turquoise module, was particularly noteworthy due to its significantly higher expression levels in soybean seeds. Data from the SoyBase database further confirmed that the *GmZF-HD1* gene is primarily expressed in the shoot apical meristem, flowers, and seeds of soybean. Given these findings, *GmZF-HD1* was chosen for subsequent validation using the yeast one-hybrid assay (Y1H).

**Figure 7:**
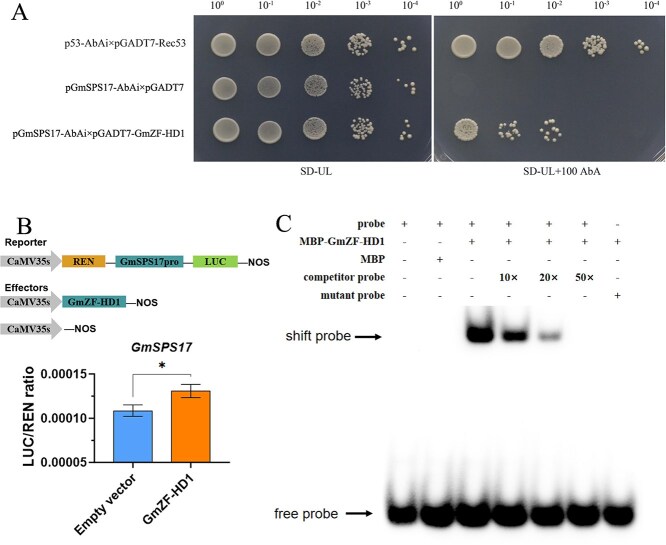
Regulation of *GmZF-HD1* on *GmSPS17* expression. (A) Yeast one-hybrid assay showing the interaction of *GmZF-HD1* with the *GmSPS17* gene promoter. The co-transformation combinations p53 + p53 promoter and pGADT7 empty vector + GmSPS17 promoter were used as the positive and negative controls, respectively. SD-UL represents the synthetic dropout medium lacking uracil and leucine (SD/-Ura/-Leu), while SD-UL + 100 AbA denotes the same medium supplemented with the minimal AbA (Aureobasidin A) inhibitory concentration of 100 ng/mL. (B) Dual-luciferase reporter assay showing the transcriptional activation of *GmZF-HD1* via the *GmSPS17* promoter. A schematic diagram of the effector and reporter constructs used for the dual-luciferase assay is shown. Values are presented as mean ± SD of three replicates; ^*^ indicates *P* < 0.05 by Student’s *t*-test. (C) EMSA showing the binding of *GmZF-HD1* to the promoter of *GmSPS17*. MBP refers to the maltose-binding protein tag.

The Y1H produced definitive results, as evidenced by the emergence of distinct colonies on SD/-Ura/-Leu plates for all groups, which confirmed the successful transformation and reliability of the experimental setup. On the selective medium supplemented with 100 μM AbA, the transformation with the empty vector pGADT7 in pGmSPS17-AbAi competent cells failed to yield any colonies, while the positive control with the pGADT7-Rec53 plasmid in p53-AbAi competent cells resulted in substantial colony growth. This contrast served to validate the effectiveness of the assay’s negative and positive controls. Significantly, the experimental group transformed with the pGADT7-GmZF-HD1 plasmid successfully grew colonies on the SD/-Ura/-Leu + 100 AbA plates, suggesting a notable interaction. This finding points to a potential binding of *GmZF-HD1* product to the promoter region of *GmSPS17*. Moreover, electrophoretic mobility shift assays (EMSA) confirmed the binding of *GmZF-HD1* to the *cis*-elements in the promoter region of *GmSPS17* (Fig. 7A and C).

Dual luciferase (LUC) reporter assays were carried out to assess the effect of *GmZF-HD1* on the promoter activity of *GmSPS17*. Using 35S::GmZF-HD1 as effector and proGmSPS17::LUC as reporter in *Nicotiana benthamiana* leaves, the expression of the effector with the reporter constructs significantly induced the activity of the *GmSPS17* promoter compared to the control (Fig. 7B), indicating the positive regulation of the *GmSPS17* promoter by *GmZF-HD1*.

Considering the overall data, these results underscore the active role of the TF *GmZF-HD1* in regulating the expression of *GmSPS17*.

### *GmZF-HD1* promotes *GmSPS17* expression and sucrose accumulation in soybean hairy roots

**Figure 8:**
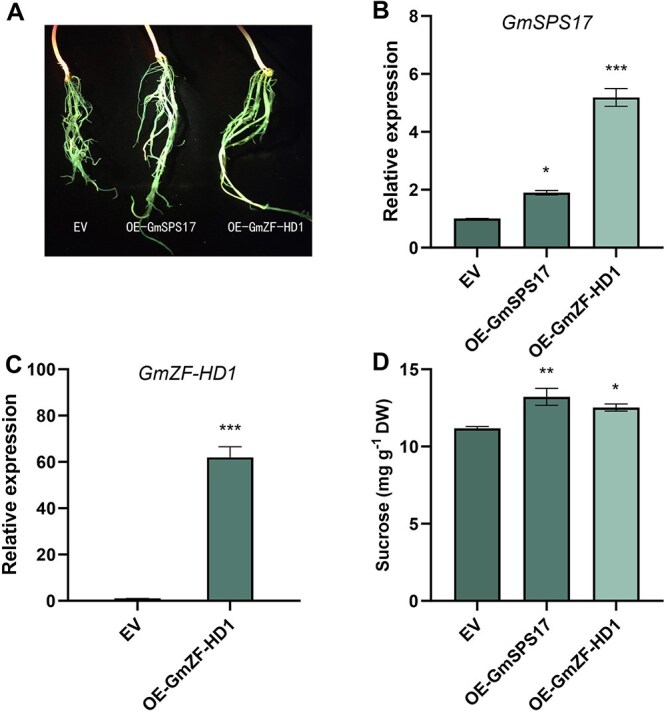
Functional validation of *GmSPS17* and *GmZF-HD1* overexpression in soybean hairy roots. (A) GFP fluorescence confirming transformation. (B, C) Relative transcript abundance of *GmSPS17* (B) and *GmZF-HD1* (C). (D) Sucrose concentration in EV control, and GmZF-HD1-overexpressing (OE-GmZF-HD1) and GmSPS17-overexpressing (OE-GmSPS17) hairy-root lines. Data are mean ± SD of six biological replicates. ^*^, ^**^, and ^***^ denote significant differences at *P* < 0.05, *P* < 0.01, and *P* < 0.001, respectively (Student’s *t*-test).

To further functionally validate the roles of *GmZF-HD1* and *GmSPS17* in sucrose accumulation, we generated transgenic hairy roots overexpressing *GmZF-HD1* (OE-GmZF-HD1) or *GmSPS17* (OE-GmSPS17). Positive transformants were selected by green fluorescent protein (GFP) fluorescence (Fig. 8A) and confirmed via qRT-PCR (Fig. 8B and C). Both overexpression lines exhibited significantly higher transcript levels of their respective target genes compared to the empty-vector (EV) control. Notably, overexpression of OE-GmZF-HD1 significantly upregulated *GmSPS17* transcription, confirming its positive regulatory role*.* These results demonstrated the successful generation of overexpression lines, enabling subsequent sucrose analysis. Quantitative analysis of sucrose content in hairy roots revealed that both OE-GmZF-HD1 and OE-GmSPS17 lines accumulated significantly higher sucrose levels compared with the EV control, with increases of 12.0% and 18.2%, respectively (Fig. 8D). These findings suggested that *GmZF-HD1* and *GmSPS17* positively regulate sucrose synthesis in soybeans.

## Discussion

### Nutritional comparison between vegetable soybeans and grain soybeans

Vegetable soybeans, prized for their enhanced flavor and larger pods relative to grain soybeans, also exhibit distinct nutritional profiles [28]. Characteristically, vegetable soybeans contain high levels of soluble sugars, protein, and free amino acids, but lower oil content [3, 29]. The present study revealed that the average 100-seed weight of vegetable soybeans was 31.9 g, significantly higher than the 18.6 g observed in grain soybeans. Considering that larger pod and seed sizes are more desirable to consumers, it is crucial to enhance these traits without adversely affecting grain yield and quality [1, 30].

Vegetable soybeans differ markedly from grain soybeans in both sensory appeal and nutritional content [31]. The sweetness of vegetable soybeans, an important factor in determining taste, is directly correlated with the levels of soluble sugars [32, 33]. Sucrose is the predominant component of total soluble sugars in soybeans [19]. In this study, vegetable soybean varieties showed 5.8% more sucrose and 9.0% more soluble sugars than grain soybeans, traits that are likely to be prioritized by breeders [34]. Consistent with the previous study [35], there was a significant upward trend of accumulation in concentrations of sucrose and soluble sugars during seed development. Additionally, it was observed that the CVs for sucrose and total soluble sugars were highest during the early pod stages and decreased as the seeds matured. This indicated complex metabolic changes in carbohydrates during seed development, and ultimately led to the stabilization of various quality components [36, 37]. Thus, understanding the accumulation and metabolism of sugars in seeds is crucial for developing high-sugar vegetable soybean varieties. For instance, the activity of SPS, an enzyme in sink tissues, has been found to significantly affect the maintenance of sucrose levels [35, 38].

The higher crude protein concentrations but lower crude oil concentrations demonstrated in both types of soybeans in the present study further support the established inverse relationship between protein and oil accumulation in soybeans [39]. Additionally, the lower level of crude oil in vegetable soybeans can be attributed to the higher 100-seed weight observed in this study, which suggests that seed size predominantly influences oil-related quality attributes [40].

Grain soybeans have higher levels of SFAs (palmitic acid + stearic acid), compared to vegetable soybeans. In contrast, vegetable soybeans are richer in UFAs (oleic acid + linoleic acid + linolenic acid), with oleic acid being especially prominent before seed maturity. These UFAs are of higher quality and are more easily absorbed by the human body [41, 42]. Oleic acid, in particular, has strong oxidative and thermal stability, which can improve the shelf life of soybean oil [43]. However, as seeds mature and approach the maturity stage, the levels of oleic acid generally decrease, likely due to its conversion into linoleic acid by desaturases regulated by genes such as *GmFAD2* [44]. This suggests that while vegetable soybeans are rich in beneficial UFAs when at the fresh edible stage, this advantage may not be preserved when the seeds mature and are utilized as grain soybeans.

Overall, compared to grain soybeans, vegetable soybeans tend to have larger seeds, higher levels of soluble sugar and protein, and a higher proportion of UFAs at immature stages but lower crude oil concentration.

### Distinguish vegetable soybeans from grain soybeans: sucrose and regulation

The distinct and discernible profiles of nutritional components at both R6 (immature pod) and R8 (full maturity) stages as indicated by PCAs underscore the nutritional differences between vegetable soybeans and grain soybeans. The question arises: beyond seed size, which specific nutritional component could serve as a pivotal biomarker to differentiate vegetable soybeans from grain soybeans? Among the 12 nutritional components determined at the R6 and R8 stages, the present study using random forest algorithm consistently identifies sucrose as the primary indicator for distinguishing vegetable soybeans from grain soybeans. This finding is aligned with the current understanding that sucrose is crucial for evaluating the edible quality of vegetable soybeans [45] and highlights its significance in the selection of vegetable soybean cultivars [31, 46].

Sucrose, a disaccharide composed of glucose and fructose, is recognized for its stable structure and pleasant sweetness [47]. Given its pivotal role in differentiating vegetable soybeans from grain soybeans, the accumulation and metabolism of sucrose in seeds should be a key focus in breeding vegetable soybean varieties. In this study, transcriptional analysis across seven soybean varieties with varying sucrose concentrations at three developmental stages identified two highly correlated modules, turquoise and yellow modules, positively correlated with sucrose concentration. These modules contained 27 candidate genes involved in sucrose metabolism, including two key enzymes: SPS and SUS. These enzymes are particularly important regulators of sucrose accumulation in soybean seeds [47, 48].

Notably, the gene encoding SPS, *GmSPS17* (also known as *GmSPSA-2*), was highly expressed in soybean roots and seeds. Previous studies have shown that overexpression of *GmSPS17* in *Arabidopsis* and soybean hairy roots revealed a significant enhancement in root starch consumption and an increase in root length and fresh weight [48, 49]. In our study, we analyzed the dynamic expression of *GmSPS17* in the seeds of 20 soybean varieties and found that its expression was significantly positively correlated with sucrose content. The overall expression level of *GmSPS17* was higher in vegetable soybeans than in grain soybeans, although the difference was not significant. This elevated expression level may be associated with variations identified in the promoter region; however, further reliable experiments are needed to confirm this hypothesis.

Another key enzyme identified among the 27 candidate genes is SUS, which can both regulate sucrose synthesis and catalyze its breakdown [35]. During the early stages of seed development, SUS primarily functions in the breakdown direction, while in the later stages, it shifts toward synthesis [35]. We further analyzed the expression of the SUS family candidate gene, *GmSUS15*, and found that its expression significantly increased in the later stages of seed development and was positively correlated with sucrose content, although the correlation coefficient was lower than that for *GmSPS17*. Therefore, we focused more on the function of *GmSPS17*.

### *GmZF-HD1*-mediated regulation of *GmSPS17* and its impact on sucrose accumulation

The distinct tissue-specific expression patterns of SPS family genes suggest that they may have unique functions [50, 51]. It is therefore essential to identify the *SPS* genes that are predominantly expressed in soybean seeds. A phylogenetic analysis of the SPS gene family in soybeans has revealed that *GmSPS17* is highly homologous to *Glyma.13G161600*, with both genes being highly expressed in soybean seeds and belonging to the *Arabidopsis AtSPS1F* family [49, 52]. Since the expression of the *GmSPS17* gene is significantly correlated with sucrose accumulation in soybean seeds, and *Glyma.13G161600* was not detected in the WGCNA module, *GmSPS17* is likely a key regulator of sucrose storage in soybean seeds.

Employing the yeast one-hybrid screen system [53] to screen for TFs, we identified 56 unique protein-encoding genes, including *GmZF-HD1*, which encodes a Zinc Finger Homeodomain (ZF-HD) TF and is implicated in the WGCNA sucrose significant correlation module. The regulatory role of *GmZF-HD1* on the *GmSPS17* promoter is confirmed by a series of complementary experiments, including subsequent yeast one-hybrid assays (Y1H), dual luciferase (LUC) reporter assays, and EMSA. They consistently demonstrated that *GmZF-HD1* activated the promoter region of *GmSPS17*, thereby driving its transcriptional expression. *GmZF-HD1*, a gene with unverified functions [54], is primarily expressed in the shoot apical meristem, flowers, and soybean seeds.

Using the *Agrobacterium rhizogenes*-mediated overexpression system, we demonstrated that overexpression of *GmZF-HD1* significantly upregulated *GmSPS17* expression, leading to enhanced sucrose accumulation in soybean hairy roots. Similarly, direct overexpression of *GmSPS17* also resulted in increased sucrose levels. These findings provide the first evidence confirming the regulatory role of *GmZF-HD1* in transcriptionally activating *GmSPS17*, thereby promoting sucrose accumulation. This insight significantly advances our understanding of the molecular mechanisms underlying sugar accumulation in soybean seeds, highlighting potential targets for improving soybean yield and quality through genetic manipulation.

## Conclusions

This study demonstrates that vegetable soybeans have higher levels of sucrose, soluble sugars, and crude protein, as well as lower crude oil and TFA levels compared to grain soybeans. Additionally, vegetable soybeans have a higher proportion of UFAs, particularly oleic acid at the fresh edible stage. These nutritional components, along with larger seed size, effectively differentiate vegetable soybeans from grain soybeans both at the fresh pod harvest stage (R6) and the mature stage (R8). Sucrose is identified as a critical marker for this distinction. *GmSPS17*, a predominant SPS gene expressed in soybean seeds, plays a key role in regulating sucrose accumulation within the sucrose regulatory modules. *GmZF-HD1* can activate *GmSPS17* transcriptional expression and modulate soybean sucrose levels.

## Materials and methods

### Experimental materials and design

A field experiment was conducted at the Agronomy Farm of the Northeast Institute of Geography and Agroecology, Chinese Academy of Sciences, Harbin. Ten vegetable soybean varieties and 10 grain soybean varieties with similar maturity were selected as experimental materials (Supplemental Supporting Information Dataset S9). All varieties were planted in a randomized complete block design with three replications. Each plot consisted of five rows with 0.65-m spacing and 5.0-m length. A uniform fertilizer application of 150 kg/ha diammonium phosphate, 20 kg/ha urea, and 60 kg/ha potassium sulfate at seeding was applied. Appropriate pesticides were used to control weeds, diseases, and insects. All varieties were sown with a rate of 280 000/ha on 1 May 2022. Samples were collected at the following stages: S1 (5 days after R5, beginning seed stage), S2 (10 days after R5), S3 (15 days after R5; R6, full seed stage), S4 (20 days after R5), and S5 (R8, full maturity stage). The harvest time was based on the classification of Fehr and Caviness [55].

### Determination of nutritional components

The crude protein concentration was determined using the combustion nitrogen analysis method with an Elementar-Vario (Elementar Analysensysteme GmbH E-III, Germany) [45]. The crude oil concentration was determined using the Soxhlet extraction method [45]. Fatty acid analyses were performed by gas chromatography (GC) using a Nexis GC-2030 (Shimadzu Corporation, Japan) equipped with a flame ionization detector, following the protocol described by Qin *et al.* [56]. Total soluble sugar and sucrose concentrations were determined by the anthrone-sulfuric acid method [33].

### cDNA library construction and RNA-Seq

A total of 63 RNA samples, belonging to seven soybean varieties at S2, S3, and S4 stages, were selected to construct the cDNA library. The library construction and sequencing were performed by Majorbio Biopharm Technology Co., Ltd (Shanghai, China) using Illumina HiSeq 4000. The data were analyzed on the online platform of the Majorbio Cloud platform (www.majorbio.com), including gene function annotation, TF prediction, and WGCNA analysis, among others [26, 57].

### Screening of a yeast one-hybrid (Y1H) cDNA library

To identify the TFs regulating *GmSPS17* expression, Y1H library screening was conducted by Biorun Biosciences Co. Ltd (Wuhan, China) using a normalized cDNA library of Williams 82. For bait plasmid construction, the promoter fragment of *GmSPS17* was cloned into the pAbAi vector and introduced into the yeast strain Y1HGold according to the manufacturer’s instruction (Clontech, USA). The positive bait recombinant clones were detected by PCR using a pair of primers GmSPS17-F and the universal primer YSH-R, yielding a fragment size of 2479 bp. The recombinant bait reporter yeasts were transformed with the prey library plasmids and then grown at 30°C for 3 to 5 days on synthetic dextrose (SD)/-Leu-Ura plates containing aureobasidin A (AbA) at a concentration of 100 ng/ml. This concentration was determined to be the minimum inhibitory concentration for the experimental group pGmSPS17-AbAi, as it completely inhibited yeast growth while allowing for the identification of interacting proteins. To validate the positive interaction, positive clones from plates of Y1H library screening were retransferred on SD/-Leu-Ura medium containing AbA. PCR was performed to amplify prey library inserts and validate the screening process using universal primers YSH-F and YSH-R. These primers were designed to amplify a 1771-bp band in the positive control to confirm the presence of the target sequence. Primers are listed in Supporting Information Dataset S10. The 1771-bp band serves as a positive control indicator, ensuring that the PCR reaction conditions are optimal and that the primers are functioning correctly. Finally, the library plasmids from positive single colonies were extracted using Yeast Plasmid DNA Kit from Tiangen Biotech (Beijing, China) Co., Ltd, for DNA sequencing, and the colonies harboring sequences encoding TFs were selected as candidates for further analyses.

### Y1H analysis of interaction

To validate the interaction between TF *GmZF-HD1* from the screening library and the *GmSPS17* promoter, the open reading frame (ORF) of *GmZF-HD1* was amplified and then fused in-frame with the GAL4 activation domain of the pGADT7 vector, resulting in the prey construct pGADT7-*GmZF-HD1*. Primers are listed in Supporting Information Dataset S10. The recombinant plasmid pGmSPS17-AbAi constructed in previous section was used as the bait construct. Plasmid pairs of pGADT7-GmZF-HD1 and pGmSPS17-AbAi were introduced into yeast strain Y1HGold following the detailed procedure of the Matchmaker Gold Yeast One-Hybrid Library Screening System (Clontech), and then grown at 30°C for 3 to 5 days on SD/-Leu-Ura plates containing aureobasidin A (AbA) at a concentration of 100 ng/ml.

### Electrophoretic mobility shift assays

The LightShift Chemiluminescent EMSA Kit (No. 20148, Thermo Fisher Scientific) was employed. The GmZF-HD1 protein, which was fused with a maltose-binding protein (MBP) tag to facilitate purification, was expressed in *Escherichia coli* strain BL21(DE3). The 5′ end of the *cis*-element probe was biotin labeled as detailed in Supporting Information Dataset S10. Competitors were synthesized via PCR and utilized in the gel-shift assay. The biotin-labeled probe was incubated with the protein extract at room temperature for 30 minutes. Subsequently, the entire reaction mixture was subjected to electrophoresis on a nondenaturing 6% polyacrylamide gel using 0.5× Tris-Borate-EDTA (TBE) buffer at a constant voltage of 60 V for 1 hour at 4°C. After electrophoresis, the gel was transferred onto a Biodyne^®^ B nylon membrane (Pall Corporation). The signals were visualized using the reagents provided in the LightShift Chemiluminescent EMSA Kit (Thermo Fisher Scientific, USA) and detected with the ChemiDoc XRS system (Bio-Rad Laboratories, USA).

### Dual-luciferase assay

The full-length coding sequence of GmZF-HD1 was cloned into the pGreenII-62SK vector to create the effector construct, while the promoter sequence of *GmSPS17* was inserted into the pGreenII 0800-LUC vector to serve as a reporter. The primers used for these constructions are listed in Supporting Information Dataset S10. The constructed reporter and effector plasmids were then introduced into tobacco (*N. benthamiana*) protoplasts via polyethylene glycol-mediated transfection. After 1 day of transient transfection, the *Renilla* (REN) and luciferase (LUC) activities were measured using a Dual-Luciferase Reporter Assay System (Vazyme, Nanjing, China). The empty pGreenII-62SK vector was used as a control. The relative LUC activity is presented as the ratio of LUC to REN values, determined from two independent biological replicates.

### Promoter amplification and sequence alignment of GmSPS17

DNA was extracted from tender leaves of 32 soybean germplasms using the NanoMagBio^®^ Plant DNA Extraction Kit (NanoMagBio Technology Co., Ltd, Wuhan, China). The promoter region of *GmSPS17* (2 kb) was amplified using specific primers (Supporting Information Dataset S10). PCR was performed with an initial denaturation at 98°C for 2 minutes, followed by 35 cycles of 98°C for 15 seconds, 58°C for 10 seconds, and 72°C for 1.5 minutes, with a final extension at 72°C for 5 minutes. The amplified fragments were purified and sequenced using the Sanger method with a BigDye Terminator v3.1 Cycle Sequencing Kit (Applied Biosystems, Foster City, CA) on an ABI 3730xl DNA Analyzer. Sequences were aligned using MEGA 6 to identify polymorphisms. *Cis*-acting elements were predicted using PlantCARE [58]. Sequence logos were generated using WebLogo (https://weblogo.threeplusone.com/).

### Quantitative reverse transcription PCR

Total RNAs were extracted using the NanoMagBio^®^ Plant RNA Kit (NanoMagBio Technology Co., Ltd, Wuhan, China), and First-Strand cDNA was synthesized using the SureScript^™^ First-Strand cDNA Kit (GeneCopoeia, Inc., Rockville, MD). qRT-PCR reactions were performed in the LightCycler^®^ 480 System with the BlazeTaq^™^ SYBR Green qPCR Mix 2.0 (GeneCopoeia) following the manufacturer’s instructions. The relative expression levels of each gene were calculated using the 2^−ΔΔCt^ analysis method. *GmActin* (*Glyma.08G146500*) was used as an internal reference gene for qRT-PCR analysis. All experiments were performed with three biological replicates, each with three technical replicates. The primers used for qRT-PCR assay are shown in Supporting Information Dataset S10.

### Generation of transgenic hairy roots

The coding sequences of *GmSPS17* and *GmZF-HD1* were cloned into the pCAMBIA1302 vector to create the pCAMBIA1302::GmSPS17 and pCAMBIA1302::GmZF-HD1 constructs. The empty pCAMBIA1302 vector was used as a control. To generate transgenic soybean hairy roots, *A. rhizogenes-*mediated transformation was performed according to the method described by Matthews and Youssef [59]. Briefly, low sucrose soybean (cv. DS707) seeds were surface sterilized and pregerminated on sterilized germination paper. The cotyledon hypocotyls of 7-day-old seedlings were cut at an angle with a sharp razor blade, and the cut surfaces of the excised seedlings were immediately infected with *A. rhizogenes* strain K599 harboring the indicated constructs (pCAMBIA1302::GmSPS17, pCAMBIA1302::GmZF-HD1, and empty pCAMBIA1302 as a control). Transformed plants were transplanted to vermiculite and cultured in a growth chamber under controlled conditions with a photoperiod of 12 hours light and 12 hours darkness for approximately 2 weeks. Positive hairy roots were verified using GFP fluorescence (Exc 440 nm, Em 500 nm, LUYOR-3415RG, China) and qPCR analysis. Concurrently, aliquots of the selected positive hairy roots underwent thermal deactivation at 105°C for 30 minutes to denature enzymes, followed by desiccation at 60°C to constant mass for sucrose determination.

### Statistical analyses of phenotypic data

Student’s *t*-test, PCAs, and correlation analyses were employed to assess the variations in different parameters between vegetable soybeans and grain soybeans, all performed using GraphPad Prism version 10.

Random forest analysis was conducted using the R package ‘rfPermute’ (Eric Archer, version 2.5), available on Zenodo (2021) [60]. All figures were created using GraphPad Prism version 10 and Adobe Illustrator CC 2019 software.

## Supplementary Material

Web_Material_uhaf242

## Data Availability

The RNA-Seq data presented in the study are deposited in the Genome Sequence Archive, accession number: PRJCA035555.
