# Supplementary material for: Sucrose as a key nutritional marker distinguishing vegetable and grain soybeans, regulated by GmZF-HD1 via GmSPS17 in seeds
Source: Hortic Res. 2025 Sep 15;12(12):uhaf242. doi: 10.1093/hr/uhaf242 (PMC12701574; doi:10.1093/hr/uhaf242)
Supplement: Web_Material_uhaf242 [file web_material_uhaf242.zip › Supplementary Figures.pdf]

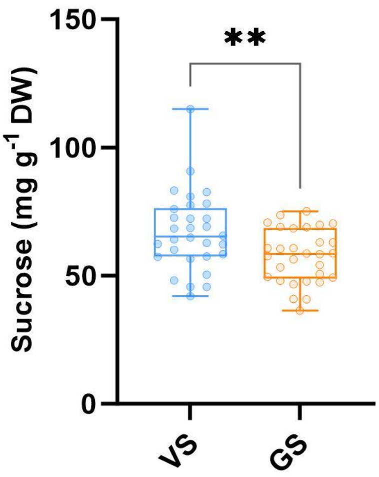

Fig. S1 Comparison of sucrose content between 30 vegetable soybean and 30 grain soybean varieties. An independent samples t-test was conducted to assess the significance of differences between the two groups, ** $P < 0.01$.

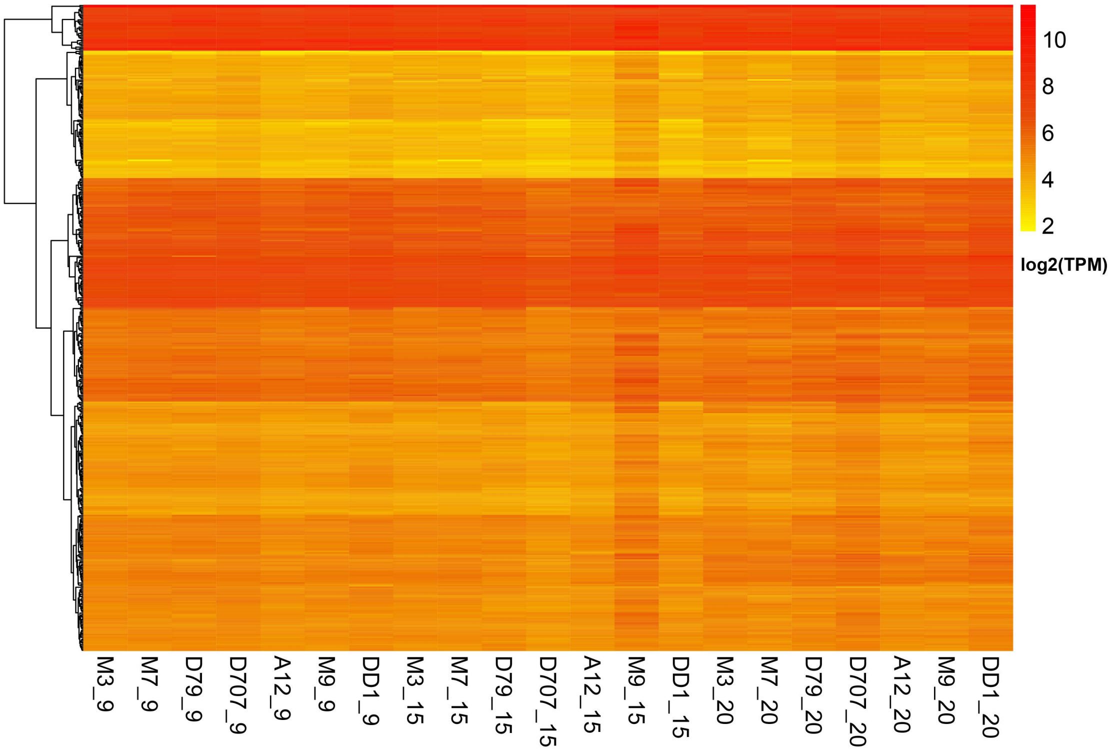

Fig. S2 The heatmap of expression pattern for 452 housekeeping genes.

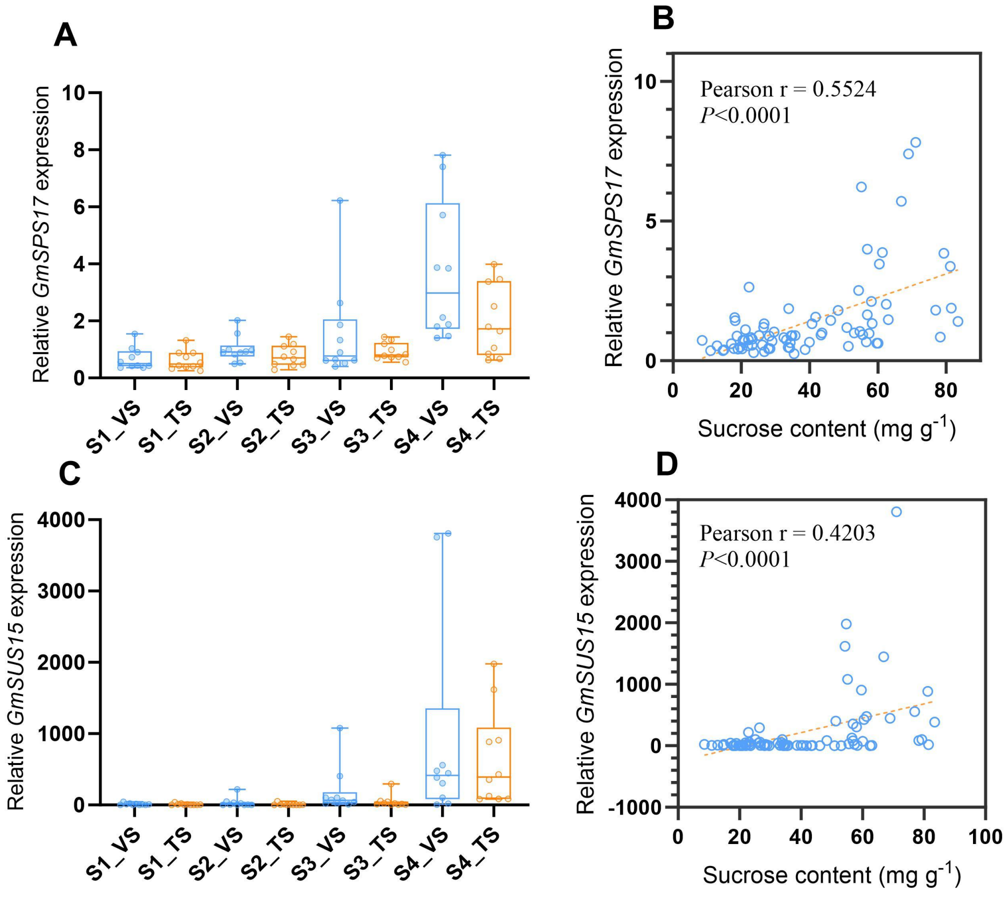

Fig. S3 Relative expression of *GmSPS17* (A) and *GmZF-HD1* (C) in seeds of 10 vegetable soybean and 10 grain soybean varieties across five developmental stages, and correlation analyses between *GmSPS17* and sucrose (B) and between *GmZF-HD1* and sucrose (D).

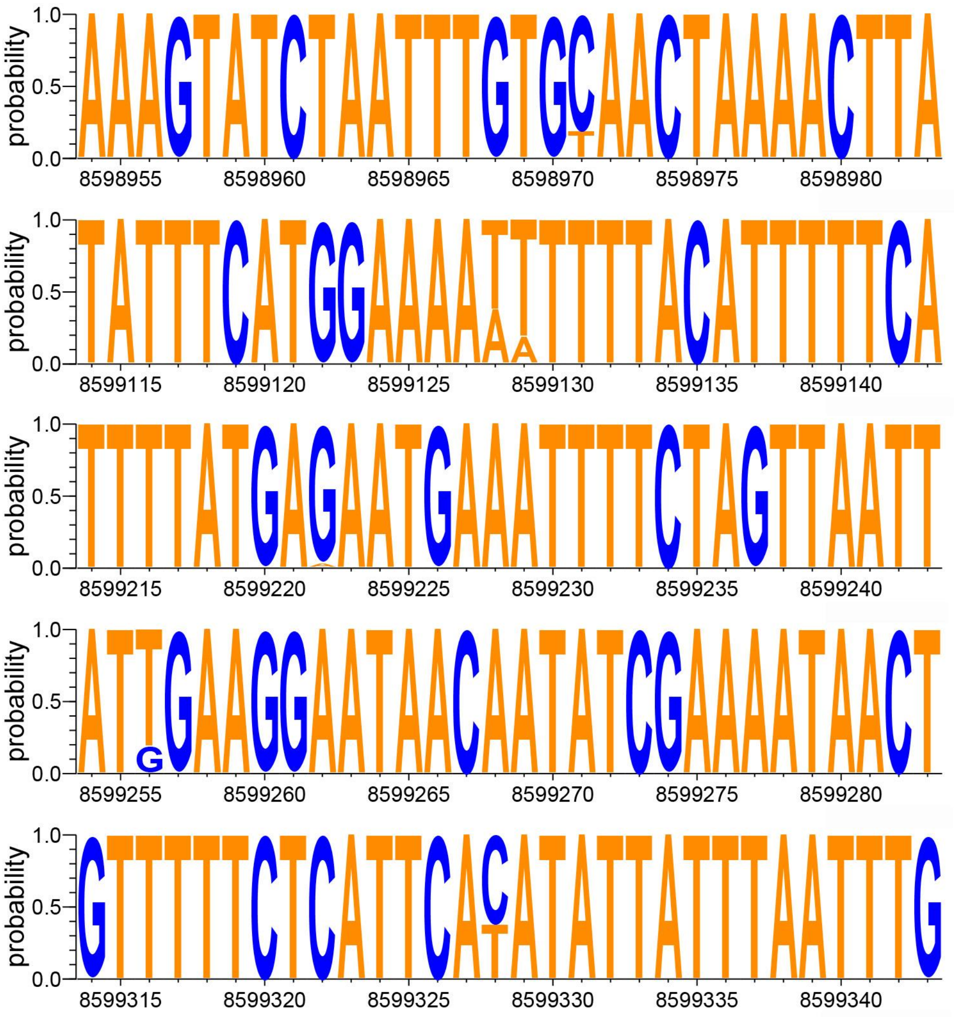

Fig. S4 Sequence alignment logo of the *GmSPS17* promoter region.
